# Salivary microbiome with gastroesophageal reflux disease and treatment

**DOI:** 10.1038/s41598-020-80170-y

**Published:** 2021-01-08

**Authors:** Nadia Kawar, Seon Gyeong Park, Joel L. Schwartz, Nicholas Callahan, Ales Obrez, Bin Yang, Zhengjia Chen, Guy R. Adami

**Affiliations:** 1 Department of Periodontics, College of Dentistry, University of Illinois at Chicago, Chicago, IL USA; 2 Department of Oral Medicine and Diagnostics, College of Dentistry, University of Illinois at Chicago, 801 South Paulina Street, Chicago, IL 60612 USA; 3 Department of Oral and Maxillofacial Surgery, College of Dentistry, University of Illinois at Chicago, Chicago, IL USA; 4 Department of Prosthodontics & Implant Innovation, College of Dentistry, University of Illinois at Chicago, Chicago, IL USA; 5 Division of Epidemiology and Biostatistics, School of Public Health, University of Illinois At Chicago, Chicago, IL USA; 6 Biostatistics Shared Resource Core, University of Illinois Cancer Center, Chicago, IL USA

**Keywords:** Microbiology, Diseases, Medical research, Pathogenesis

## Abstract

The effect of oral microbial composition on periodontal health and on systemic health has been, and is being established. The oral microbiome, in turn, can be altered by local and systemic diseases and conditions. Gastroesophageal reflux disease (GERD), has been associated with increased acidity in the oral cavity resulting in dental erosion, and controversially a reduced risk of periodontal disease. We hypothesized that presence of GERD was linked to a modified microbial profile in untreated GERD patients and that the use of proton pump inhibitor (PPI) drugs: potent disruptors of gut microbiome, in GERD patients might result in a salivary microbiome that is further distinct. Untreated GERD patients showed multiple differences in salivary microbiome as compared to healthy controls. Taxa found at lower levels related to the presence of GERD not treated by PPI included: *Prevotella melaninogenica, Prevotella pallens, Leptotrichia*, and *Solobacterium moorei and thirteen others*. In contrast, GERD patients chronically using PPI showed minimal differences in salivary taxa compared to healthy controls not using PPI.

## Introduction

There are hundreds of species of bacteria that inhabit the mucosal and hard surfaces of the oral cavity, and upper respiratory and gastrointestinal tracts, that can be sampled by examining the saliva^1,2^. Oral bacteria of the mouth have been shown not only to play a role in oral disease such as caries, dental erosion, periodontal disease, and mucosal lesions, but also may contribute to systemic disease such as cardiovascular disease, diabetes mellitus, even pancreatic cancer^1,3–5^. A large number of bacteria genera and species contribute to a complex oral microbial ecosystem, thus it is difficult to differentiate a healthy microbiome or what used to be described as “normal oral flora” versus a pathogenic oral microbiome. A first step is identifying specific bacteria taxa that are associated with a disease or condition.

Gastroesophageal reflux disease (GERD) is a condition that is believed to occur due to dysfunction of the lower esophageal sphincter, which is responsible for preventing back flow of stomach contents into the esophagus^6^. This regurgitation of gastric juices into the esophagus, oropharyngeal and oral cavity lowers their pH^7^. In the esophagus, this can contribute to chronic inflammation, histological changes in the mucosal cells lining the distal esophagus, and increased risk for esophageal cancer^8,9^. Changes in distal esophageal microbiome have been characterized, including lower levels of Prevotella, Helicobacter, and Moraxella genera with GERD^10–12^. In the oral cavity, the presence of GERD is associated with acidic saliva (pH 4.9) versus the near neutral pH of 6.5 in healthy subjects^13^. Inflammation of the oral mucosa is often seen, as well as reduction in oral salivary flow^14,15^. This acidic environment favors loss of tooth structure through enamel erosion.

PPIs are drugs that work by inhibiting release of Hydrogen ions from mucosal cells of the GI tract, particularly those in the stomach^7^. While PPIs are indicated for usage against H. Pylori infection, erosive esophagitis, gastric ulcers, and for stress ulcer prevention in critically ill patients, they are available over the counter and are used long term for a number of other aliments^9,16,17^. Epidemiological studies reveal that usage of PPIs is associated with increased risk of *Clostridium difficile* infection in the gut, small intestinal bacterial overgrowth, pneumonia, and nutritional deficiencies^18–20^. These illnesses are thought to be linked to reduced levels of gastric acid release and increased pH in stomach and small intestine as well as other sites. PPI usage can have effects on upper GI sites such as esophagus and oral cavity even in healthy subjects. After 4 week of PPI administration, healthy subjects were seen to show changes in salivary microbiota, including reductions in *Neisseria, Veillonella*, possibly *Haemophilus*, and an increase in *Streptococcus*^21^.

The aim of this study was to determine the role of GERD in the makeup of the oral microbiome. This study examined the salivary microbiome in dental patients who reported with GERD. In addition, the salivary microbiome of patients with GERD chronically treated with PPI was also analyzed. In patients with GERD, usage of PPI has been shown to be associated with restoration of normal pH and possibly saliva flow^7^. In these patients one might expect the oral microbiome to be more similar to healthy subjects.

## Results

### Selected subjects

In the original study samples were collected from 208 subjects who came to the dental clinics for checkups***. ***Of these 36 self-reported having GERD comprised of 20 patients with GERD who used PPI (GERD + PPI) group, and 16 who had GERD but did not use medication (GERD + No PPI) group. Negative controls did not have GERD and did not use PPI. This negative control group had a lower percentage of tobacco usage, fewer females and trended toward a higher level of periodontal disease than the group with GERD that did not use PPI (Supplemental Table 1).

To facilitate comparison between the GERD subjects that did not use PPI and the negative control a subset of 92 negative control subjects was created so to minimize differences in clinical covariates. There was a total of 128 subjects in total in the final groups used for saliva microbiome analysis in this study (Table 1). Sixty patients were edentulous.

**Table 1:** Demographic and clinical indices after matching.

Feature		Control	GERD no PPI		GERD + PPI		Control + PPI	
Sex^1^	Female	54	14		13		4	
	Male	38	2	p < 0.028	7	*p* < 0.629	6	p < 0.346
Age^2^	Mean	57.8 ± 1.66	54.4 ± 4.0	p < 0.367	63.5 ± 2.2	p < 0.120	67.4 ± 3.5	p < 0.047
Tobacco User^1^	User	31	7		7		2	
	Nonuser	61	9	p < 0.571	13	p < 0.419	8	p < 1
Dentate^1^	No	21	4		6		8	
	Yes	71	12	p < 1.0	14	p < 1	2	p < 0.0009
Periodontal Disease^1^	No	80	14		13		8	
	Yes	12	2	p < 1.0	7	p < 0.127	2	p < 0.500

^1^Fisher Exact Test versus control.

^2^Student *t* Test versus control.

^3^Periodontal disease is Class III and IV by ADP/AAP.

### Comparison of microbial communities among GERD patients with and without PPI usage

**Figure 1:**
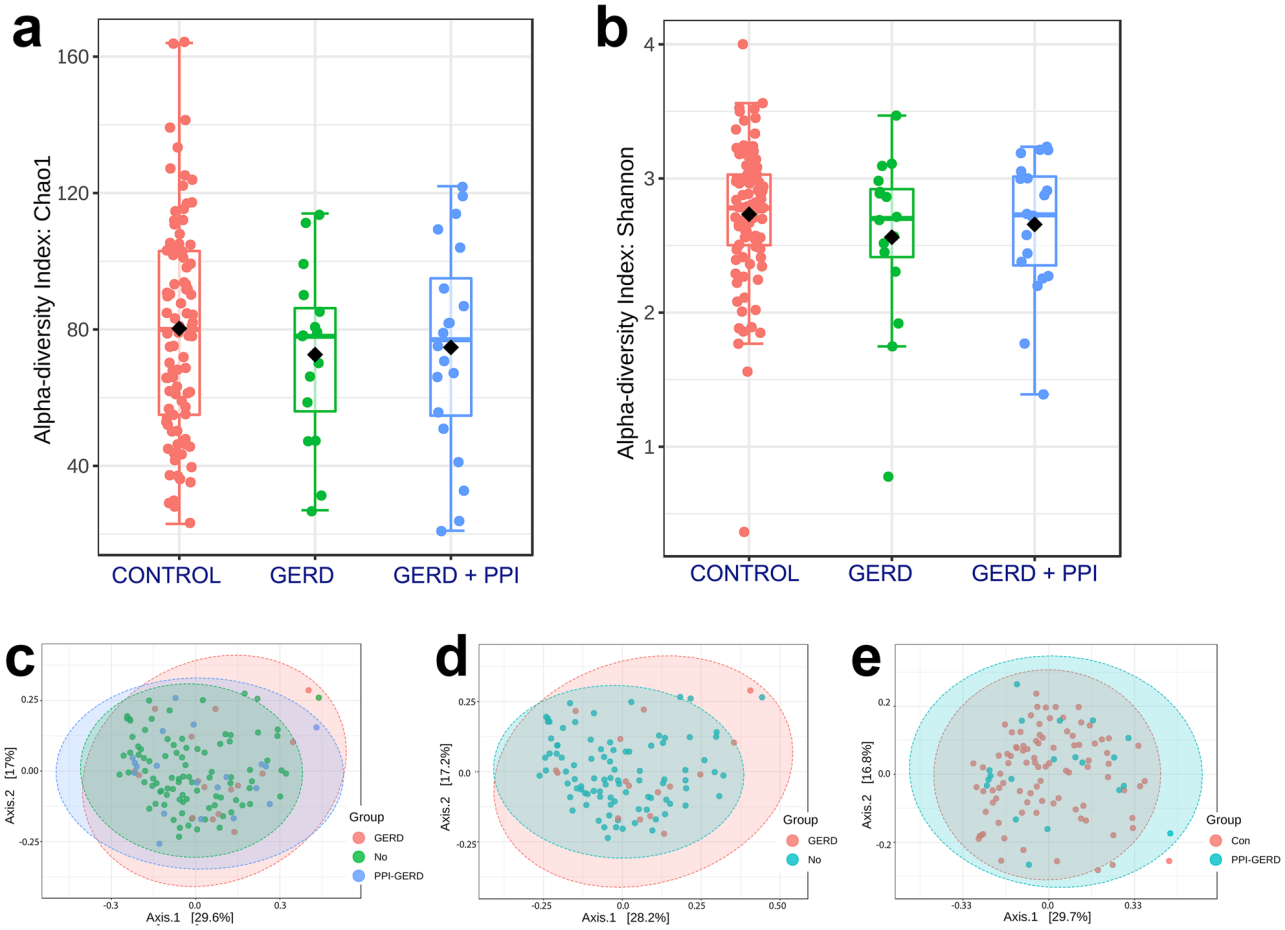
Box plot of (**a**) Chao1 index of control, GERD patients not using PPI, and GERD patients using PPI. Comparison of the control to GERD patients not using PPI, *p* < 0.302, and the control comparison to GERD using PPI, *p* < 0.513; and (**b**) Shannon index in controls versus GERD patients not using PPI, *p* < 0.330 and versus GERD using PPI, *p* < 0.571. Principal coordinate analysis (PCoA) of saliva microbiome profiles of (**c**) all 3 groups; (**d**) negative controls versus GERD patient not using PPI; and (**e**) negative controls versus GERD patients using PPI reveal some differences in microbiota community structure in the GERD group not using PPI.

Differences in the microbial population in the large negative control group, the group with GERD that did not use PPI, and the group with GERD that did use PPI, were examined. In total 4,964,720 reads from 128 saliva samples were obtained. These were subjected to DADA2 for variant identification and followed by BLAST analysis so to be assigned to taxa of the Human Oral Microbiome Database (HOMD)^27^. Alpha diversity analysis revealed no significant differences among the groups (Fig. 1a,b). For beta diversity study, overall bacterial community structure was compared between the negative controls and the two other groups using Bray Curtis analysis (Fig. 1c–e). Principal coordinate analysis (PCoA) with Bray Curtis to calculate distances between the negative controls and GERD patients not using PPI showed the groups fell into clusters with some subtle differences. Due to the unbalanced nature of the datasets quantitation by PEMANOVA or ANOSIM of the differences was not possible^29^. A similar comparison of the GERD + PPI group to negative controls revealed the scatter plots were superimposable, and thus similar (Fig. 1e).

### Examination of individual taxa in the three groups

**Figure 2:**
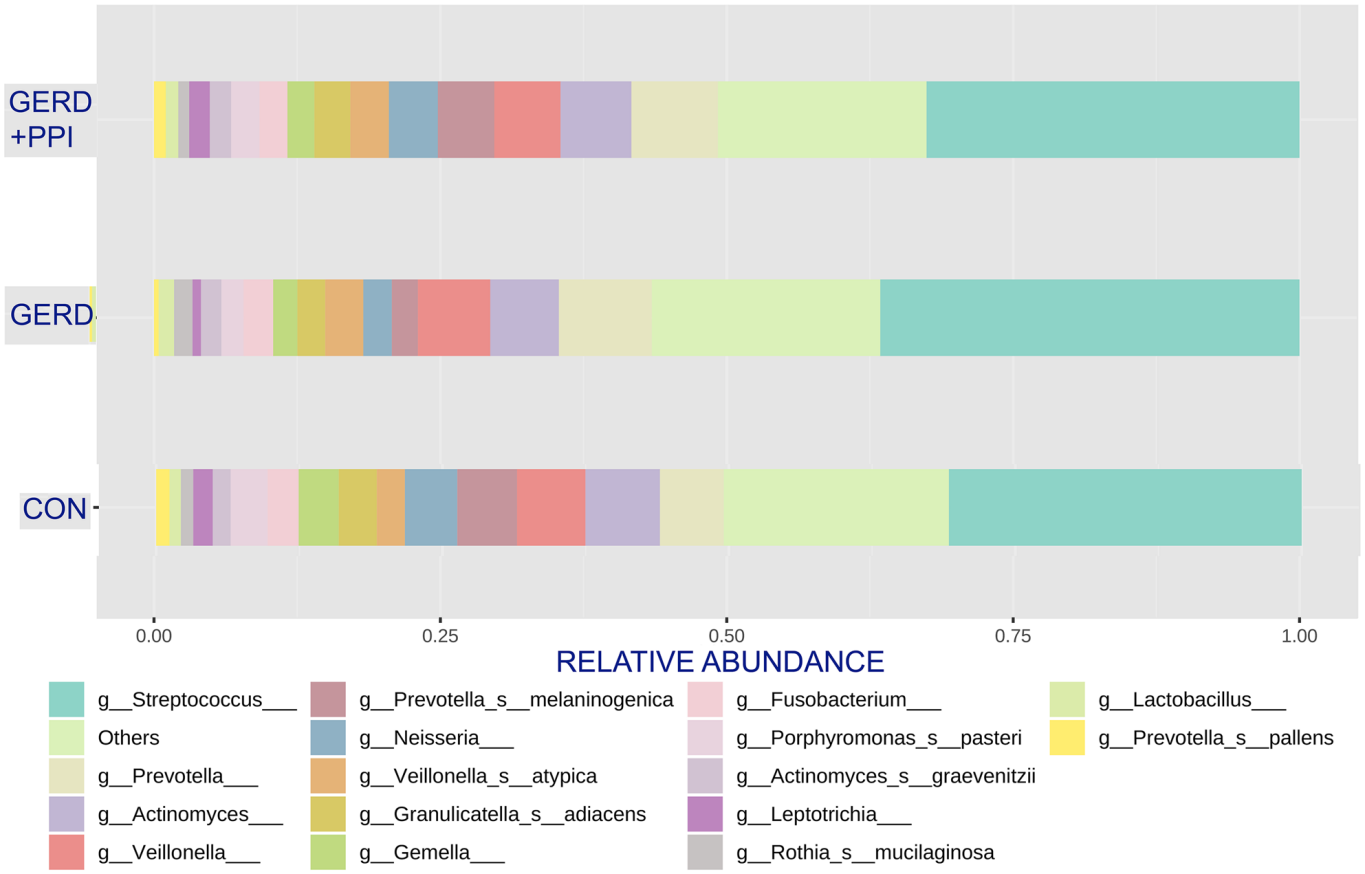
The bar plot reveals the 16 most common species and/or genus subgroups in saliva of the three groups studied (Control, without GERD and not using PPI, GERD, GERD patients not using PPI, and GERD + PPI, GERD patients using PPI) and allows comparison of relative taxa proportions.

Analysis of the 16 s rDNA based HOMD database taxonomic assignments revealed in the barplot the 16 most common species and genera in GERD patients not using PPI and those found in the negative controls without GERD and not using PPI (Fig. 2). Abundant taxa that were significantly different in the two groups included *Prevotella pallens, Prevotella melaninogenica and Leptotrichia* (see below). Figure 2 also displays the distribution of major taxa for the GERD + PPI patients. The distribution of the major taxa looks remarkably similar to the controls without GERD and not using PPI.

**Figure 3:**
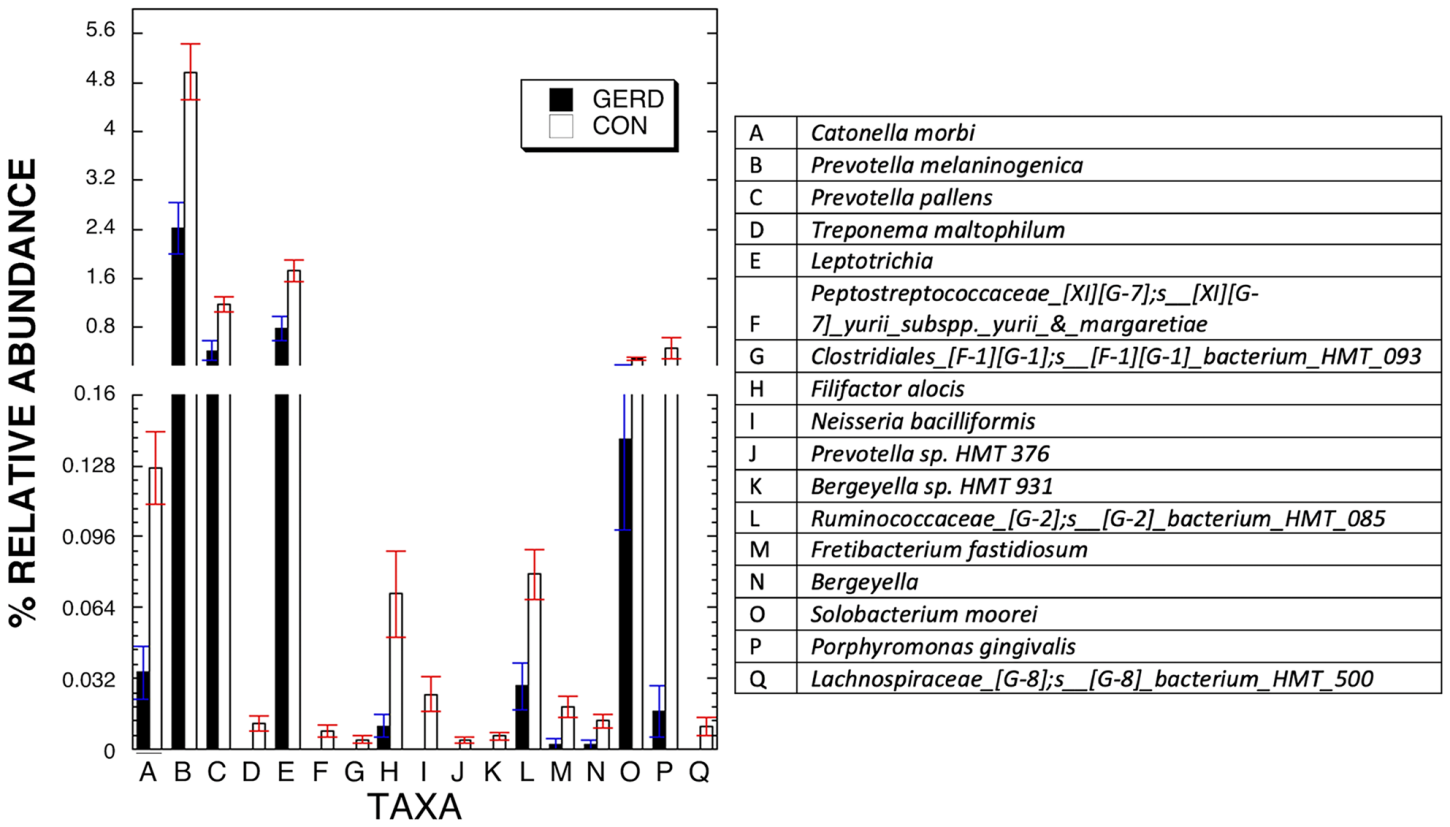
Differentially abundant species in saliva of GERD patients not using PPI versus negative controls without GERD and not using PPI. Shown are the 17 taxa that showed changes in relative abundance at FDR < 0.05 in separate analyses V3 16S rDNA. Bars represent relative abundance in the two groups.

A key question is which of the apparent differences between the GERD with no PPI group, and the negative controls, were statistically significant. Two sample Welch’s *t*-test which is robust for imbalance of sample size between groups was employed to determine the specific difference between the two groups^31^. Of 176 taxa at the species and genus subset level in the saliva of the three patient groups, 17 were shown by Welch’s *t*-test, to be at different levels in the group of patients with GERD who did not use PPI versus the controls, FDR < 0.1 (Fig. 3). The same comparison of subjects with GERD using PPI versus the negative controls gave a different result. No taxa came close to showing a difference in levels between the two groups at FDR < 0.2.

While the GERD subjects not using PPI and negative controls were matched based on periodontal status, edentualism, and more or less for tobacco use, they still differed by gender. For that reason, multivariable analysis was used to re-examine the differences between this group and the controls after adjusting for differences in gender and tobacco usage in the two groups. Table 2 indicates that after adjustment for gender and tobacco 4 taxa were revealed to be significantly different between the two groups.

## Discussion

This observational study is the first that we know of to characterize the salivary microbiome in GERD patients using PPI and in those with GERD who do not use PPI. Within the original population in this study, subjects who had GERD and did not use PPI were more frequently tobacco users and trended toward having no, or milder, periodontal disease (Supp. Table 1). Along with GERD patient groups a matched group negative control without GERD who did not use PPI was further analyzed for salivary microbiome. Even after matching for age, periodontal status, and edentualism, which may affect salivary bacteria^1,32^, there were 17 species and subgenera that were at different levels in the GERD patients not using PPI compared to negative controls. Four of the 17 bacteria taxa shown to be decreased in the GERD patients who did not use PPI compared to controls, *Porphyromonas gingivalis, Filifactor alocis, Fretibacterium fastidiosum*, and *Lachnospiraceae_[G-8];s_[G-8]_bacterium_HMT_500,* have been found to be elevated in the saliva and/or periodontia of patients with chronic or aggressive periodontitis (Fig. 3)^33–35^. This is consistent with the recent findings that GERD patients have lower risk for periodontal disease^36^. Multivariable analysis adjusting for a trend toward differences in tobacco usage, and an imbalance in gender, in the GERD group versus the negative controls, revealed none of these 4 taxa showed statistically significant differences in level. This relationship between GERD and the 4 periodontal disease-related taxa may well not be direct. Further exploration of the role of these periodontal pathogens in GERD effects on the oral cavity awaits a larger study.

Adverse effects of long-term use of PPI on other organ systems has been established and cautioned against^19,20,37^, presumably due to pH increases. By lowering acid release in the stomach, the pH there and in the intestine can be increased, which can change microbiome at those sites whether the patient is healthy or not^11,12,38–40^. Less attention has been paid to how PPI usage affects the oral or esophageal microbiota in GERD patients. Earlier it has been shown that healthy subjects without GERD who used PPIs for 4 weeks experienced changes in oral bacterial taxa^21^. In contrast, the subset of GERD patients chronically treated with PPI showed salivary taxa profiles quite similar to that of the negative controls (Fig. 1, 2). Finally, the GERD patients in this study who did not use PPI showed 17 different taxa at reduced levels versus negative controls. Four taxa, *Prevotella melaninogenica, Prevotella pallens, Solobacterium moorei,* and *Leptotrichia* remained at lower abundance in this group versus the negative control group after multivariable analysis suggesting the lower levels of these taxa in these subjects (Table 2) is directly related to the presence of GERD without PPI treatment. Together these studies revealed that these factors, GERD or PPI separately, were linked to differences in oral microbiome. When present together, the effects seem to be minimized. It has already been shown that usage of PPI can restore normal oral pH, and even salivary flow, in GERD patients^13,14^. One can speculate that in the oral cavity, a body site not normally subjected to gastric acid, the usage of a PPI to inhibit acid production in the gut would help maintain a healthy microbiota. Though not studied here it is expected to have a similar effect on the esophagus with long-term treatment of GERD with PPIs linked to a normal or healthy microbiome. While the current study included 36 subjects with GERD, the groups were small after stratification by PPI usage. It was unclear what caused the differences in salivary microbiome among the groups and whether the presence of GERD has a complex role in oral microbiome makeup. GERD patients who did not use PPI could do this for different reasons^7^. One cannot be certain that having GERD and not using PPI causes the differences in microbiome. To get a better understanding about GERD effects on oral microbiome and, in turn, PPI effects on GERD, it will be necessary to study patients before and after administration of these drugs.

**Table 2:** Summary of composition of the oral microbiome by two groups.

Microbiome	Multivariate analysis results**		
Outcome variables			
	Control	GERD	*p* value
	(N = 92)	(N = 16)	
*Catonella morbi*	0.129623	0.061025	0.086
	− 0.015568	− 0.037048	
*Prevotella melaninogenica*	4.889842	2.392873	0.031
	− 0.448333	− 1.066951	
*Prevotella pallens*	1.145807	0.308243	0.008
	− 0.121866	− 0.290018	
*Treponema maltophilum*	0.012477	− 0.000206	0.126
	− 0.003232	− 0.007691	
*Leptotrichia*	1.625858	0.690662	0.023
	− 0.159576	− 0.379762	
*Peptostreptococcaceae_[XI][G-7];s__[XI][G-7]_yurii_subspp._yurii_*&*_margaretiae*	0.006525	6.97E−05	0.308
	− 0.002479	− 0.005899	
*Clostridiales_[F-1][G-1];s__[F-1][G-1]_bacterium_HMT_093*	0.005791	0.001101	0.190
	− 0.001397	− 0.003325	
*Filifactor alocis*	0.089173	0.029482	0.193
	− 0.017922	− 0.042651	
*Neisseria bacilliformis*	0.026814	0.002862	0.230
	− 0.007802	− 0.018568	
*Prevotella sp. HMT 376*	0.003765	− 0.00108	0.135
	− 0.001265	− 0.00301	
*Bergeyella sp. HMT 931*	0.006029	0.00092	0.306
	− 0.001954	− 0.004649	
*Ruminococcaceae_[G-2];s__[G-2]_bacterium_HMT_085*	0.083247	0.031361	0.074
	− 0.011299	− 0.026889	
*Fretibacterium fastidiosum*	0.024701	0.008493	0.153
	− 0.004434	− 0.010551	
*Bergeyella*	0.0113	− 0.000885	0.119
	− 0.003053	− 0.007266	
*Solobacterium moorei*	0.297201	0.135003	0.047
	− 0.0318	− 0.075679	
*Porphyromonas gingivalis*	0.572156	0.021349	0.158
	− 0.152375	− 0.362623	
*Lachnospiraceae_[G-8];s__[G-8]_bacterium_HMT_500*	0.012903	0.001121	0.232
	− 0.003852	− 0.009168	

**The least square means and SE were estimated using general linear model (GLM) after adjusting for Gender and Tobacco User. Shown is range of values and *p* value.

## Conclusion

The differences in bacteria taxa levels in untreated GERD patients may be a result of a pH change due to GERD and may have effects on oral conditions. The long-term effects on oral health due to the differences can only be guessed. Remarkably, in GERD subjects who use PPI, the salivary microbiome was quite similar to that in negative controls without the disease not using PPI. This would suggest at least some benefit in the oral cavity with usage of those drugs in people with GERD. Clinical studies looking at the effects of GERD on the oral cavity should take into consideration salivary oral microbial differences between treated and untreated GERD patients.

## Material and methods

### Study design

A cross-sectional study was conducted at the University of Illinois at Chicago College of Dentistry, General Practice and Denture clinics that compared the oral microbiome using stimulated saliva^22^ between subjects with GERD and those without, with an effort to match subjects based on age. All subjects were questioned on physician diagnosed medical conditions on each visit as part of routine clinical procedures. Subjects who indicated that they had GERD at multiple visits including time of sample collection were considered to have GERD. Patients who included GERD in the medical history at the time of collection but no other time, and vice versa, were reinterviewed to confirm GERD diagnosis. Twelve potential subjects who used PPI but did not report as having GERD were also reinterviewed, two reported as having GERD, the remaining ten did not and were not included in the study. All subjects provided written informed consent to participate in accordance with guidelines of the Office for the Protection of Research Subjects of the University of Illinois at Chicago, with formal approval of the study protocol, 2016-0696, by the Institutional Review Board 1 of the University of Illinois at Chicago.

Study inclusion criteria were: Subjects 18 years and older; complete medical record; current medication list; and full periodontal exam. Study exclusion criteria were: presence of restored dental implants; removable partial dentures; scaling of teeth within the past 3 months; acute disease that requires urgent care; less than twenty (20) natural teeth for the dentate subjects; antibiotic use within the past month; use of antimicrobial mouthwash within the past 48 h; and food consumption within the past 1 h. Periodontal health was assessed using the ADA/AAP classification system based on clinical and radiographic findings. Subjects were classified into groups based on the severity of the disease. Classes I and II are considered healthy or have mild form of periodontal disease, Class III have moderate and Class IV have severe periodontal disease. The focus is active, not historical periodontal disease. Edentulous patients without periodontia are considered to lack active periodontal disease. For the purpose of microbiome analysis, subjects in the negative control group were selected to maximize matching with the GERD without PPI group in regard to periodontal disease status, tobacco usage, presence of teeth and age.

### Sampling procedure

After rinsing with water, patients chewed on paraffin wax for 5 min and then stimulated saliva was collected. All samples were centrifuged at 6000 × g for 5 min prior to 2× washing of pellet with cold PBS and storage frozen prior to DNA extraction^23^.

### Characterization of microbial community structure

Genomic DNA was extracted from saliva samples as described earlier^23^. Amplicon assay targeted the V1-V3 variable region of bacterial 16S ribosomal RNA rRNA genes as described^23^. The second PCR reactions with barcoding and the Illumina miSeq sequencing were performed at the University of Illinois at Chicago Sequencing Core.

### Bioinformatics analysis

For taxa assignment and measurement, reverse sequences from the FASTQ files were analyzed using the software package QIIME2^24,25^. Sequences were trimmed if the average quality was lower than 25. As a result, the read sequences were truncated at 258nt. DADA2-plugin in QIIME2 was used to sequence, denoise, and generate feature data and feature tables for the dataset^26^. Taxonomy assignments were done by classify-consensus-blast function with 98% match identity to the Human Oral Microbiome Database^27^. Of the 208 samples, there were 13,383 to 63,154 reads per sample. There were 8,349,860 reads total. Data from 3 additional subject samples were discarded due to low read numbers.

Alpha diversity analysis were performed using MicrobiomeAnalyst^28^. This analysis included calculation of Shannon’s diversity index of both species number and their distribution, and Chao1′s of richness. The significance of the differences between each group and the controls was derived using an unpaired Welch’s *t*-test. Beta diversity analysis was visulalized using Bray Curtis dissimilarity (non-phylogenetic) metric. PERMANOVA or ANOSIM test to determine differences between groups were not run due to the unbalanced datasets used^29^.

STAMP was used to determine differences in specific microbiota taxonomic abundance between the groups using Welch’s *t-*test and with determination of false discovery rate (FDR) using the Benjamini Hochberg test^30^. This was performed after eliminating taxa that appeared in 10% or fewer subjects at < 2 reads^30^.

General linear model (GLM) was used in the multivariable analyses to investigate the adjusted difference in Taxa between different groups after adjusting for significant patient’s characteristic and clinical factors. The best predictive GLM model was used to explore the significant predictors of Taxa using a backward selection with a retaining criteria of 0.2. All multivariable analyses were done in SPSS and SAS.

## Supplementary Information

Supplementary Information.

## Data Availability

The sequencing data from this study is deposited as PRJNA674379 in the National Center for Biotechnology Information Sequence Read Archive.
