# Supplementary material for: Salivary microbiome with gastroesophageal reflux disease and treatment
Source: Sci Rep. 2021 Jan 8;11:188. doi: 10.1038/s41598-020-80170-y (PMC7794605; doi:10.1038/s41598-020-80170-y)
Supplement: Supplementary file 1 — Supplementary Information. [file 41598_2020_80170_MOESM1_ESM.docx]

**Supplementary Information**

Salivary Microbiome with Gastroesophageal Reflux Disease and Treatment

Nadia Kawar, Seon G. Park, Joel L. Schwartz, Nicholas Callahan, Ales Obrez, Bin Yang, Zhengjia Chen, Guy R. Adami

| Supplemental Table 1. Demographic and clinical indices of original sample population. | | | |  |  |  |
| --- | --- | --- | --- | --- | --- | --- |
| Feature |  | Control | GERD no PPI |  | GERD+ PPI |  |
| Sex^1^ | Female | 91 | 14 |  | 13 |  |
|  | Male | 71 | 2 | p < 0.016 | 7 | p < 0.484 |
| Age^2^ | Mean | 57.3±1.2 | 54.4±3.1 | p < 0.470 | 63.5±2.2 | p < 0.484 |
| Tobacco User^1^ | User | 32 | 7 |  | 7 |  |
|  | Nonuser | 130 | 9 | p < 0.050 | 13 | p < 0.147 |
| Dentate^1^ | No | 42 | 4 |  | 6 |  |
|  | Yes | 120 | 12 | p < 1.0 | 14 | p < 0.788 |
| Periodontal Disease^1,3^ | No | 107 | 14 |  | 13 |  |
|  | Yes | 55 | 2 | p < 0.096 | 7 | p < 1.0 |
|  |  |  |  |  |  |  |
| ^1^ Fisher Exact Test versus control | |  |  |  |  |  |
| ^2^ Student t Test versus control  ^3^ Periodontal disease is Class III and IV by ADP/ AAP | |  |  |  |  |  |
